# Developments in esophageal surgery for adenocarcinoma: a comparison of two decades

**DOI:** 10.1186/1471-2407-7-114

**Published:** 2007-06-29

**Authors:** I Gockel, FS Sultanov, M Domeyer, U Goenner, Th Junginger

**Affiliations:** 1 Department of General and Abdominal Surgery, Johannes Gutenberg-University of Mainz, Mainz, Germany

## Abstract

**Background:**

The objective of this study was to examine outcomes in patients undergoing esophageal resection for adenocarcinoma at our institution during a 20-year period and, in particular, to address temporal trends in long-term survival.

**Methods:**

Out of 470 patients who underwent esophagectomy for malignancy between September 1985 and September 2005, a total number of 175 patients presented with esophageal adenocarcinoma. Patients enrolled in this study included AEG (adenocarcinoma of the esophagogastric junction) type I tumors only. Time trends were studied comparing two decades, 9/1985 to 9/1995 (DI) and 10/1995 to 9/2005 (DII).

**Results:**

The overall survival was significantly more favourable in patients undergoing esophageal resection for adenocarcinoma in the recent time period (DII, 10/1995 to 9/2005) as compared to the early time period (DI, 9/1985 to 9/1995) (log rank test: p = 0.0329). Significant differences in the recent decade were seen based on lower ASA-classifications, earlier tumor stages, and the operative procedure with a higher frequency of transhiatal resections (p < 0.05). 30-day mortality improved from 8.3% to 3.1% during the 20-year time-interval, thus without statistical significance.

**Conclusion:**

Based on our experience, overall survival is improving over time for adenocarcinoma of the esophagus. Factors that may play an important role in this trend include early diagnosis and improved patient selection through better preoperative staging, improved surgical technique with a tailored approach carefully evaluated by physiologic patient status, comorbidity and tumor extent.

## Background

Esophageal resection is the only curative therapy for patients with esophageal carcinoma. Although a variety of retrospective studies have demonstrated improvements in short-term outcomes in recent years, changes in long-term survival over time are less well-established. Advances in surgical planning, operative technique, and perioperative care have resulted in improved short-term outcomes, with experienced centers now reporting in-hospital mortality rates of less than 5%, even with major resections [1].

Long-term survival and potential for cure following surgical resection for esophageal adenocarcinoma have been demonstrated in numerous uncontrolled studies. As esophageal surgery has become safer and indications more differentiated, especially with respect to neoadjuvant therapy, there are increasing expectations regarding assessment of trends in long-term patient outcomes. Although some improvements in survival are being reported in more recent series compared with those from earlier decades, survival trends within a given group or institution have not been clearly demonstrated.

The objective of this study was to examine outcomes in patients undergoing esophageal resection for adenocarcinoma at our institution during a 20-year period and, in particular, to address temporal trends in long-term survival.

## Methods

Out of 470 patients who underwent esophagectomy for malignancy between September 1985 and September 2005 in the Department of General and Abdominal Surgery of the Johannes Gutenberg-University Hospital of Mainz, a total number of 175 patients presented with esophageal adenocarcinoma. Adenocarcinoma enrolled in this study only included AEG type I tumors [2]. Type II (tumors of the cardia) and type III (subcardial tumors with infiltration of the cardia and/or distal esophagus) were strictly not taken into consideration.

For preoperative staging, EUS (endoscopic ultrasound), CT (computed tomography) of the neck, chest and abdomen and PET (positron emission tomography) were routinely carried out. Thus, endoscopic ultrasound and PET scan were not available at the initiation of the study back in 1985.

Transhiatal esophagectomy with abdominal and posterior mediastinal lymphadenectomy was routinely carried out in adenocarcinoma, whereas the transthoracic procedure with two-field lymphadenectomy was accomplished in the presence of advanced tumor growth or extended lymph node involvement. The transhiatal procedure was done with an abdominal lymph node dissection (including the paracardial nodes, the left gastric artery nodes along with the lymph nodes of the lesser curvature of the stomach, the celiac trunc, the common hepatic artery and in selected cases – as macroscopic tumor involvement – the splenic artery), as well as with an excision of the lymph nodes extending as far as the carina of the trachea, and to those lymph nodes which could be reached in the lower, posterior mediastinum. The transthoracic technique, performed via a right dorso-lateral thoracotomy, involved an abdominal (as described) and a more extensive mediastinal lymphadenectomy in the sense of a two-field dissection. The specimen here included the lower and middle mediastinal, subcarinal, and right-sided paratracheal lymph nodes (en bloc dissection). Paratracheal and bifurcal nodes were only removed on both sides in case of clinical suspicion of bilateral involvement. The aortopulmonary – window nodes were dissected separately.

Neoadjuvant therapy was not administered in our patients with adenocarcinoma.

Deaths within 30 days of operation were considered 30-day mortality. Patient follow-up was obtained from death certificates, office records, letter or telephone contact.

Time trends were studied comparing two decades, 9/1985 to 9/1995 (DI) and 10/1995 to 9/2005 (DII). These two periods were chosen in order to achieve a balance of sufficient sample number and adequate follow-up.

### Statistical analysis

Data were collected prospectively in a specially established database and retrospectively analyzed.

The SSPS 12.0 software package was used for statistical data analysis (SSPS, Chicago, IL, USA: 2001). Data are expressed as median with ranges (minimum - maximum), or as percentages (%).

Patient demographics, operative and pathologic findings, and the postoperative course were evaluated both by univariate and multivariate models to determine the impact on overall survival, which was calculated from the time of esophageal resection. Survival analyses were estimated by the Kaplan-Meier method [3]. Differences in survival were compared using the log-rank test. Fisher's exact or the Χ-square tests were used for univariate comparisons. Multivariate analysis was performed with the Cox Proportional Hazard Model [4]. Differences were considered significant if *p *< 0.05.

## Results

### Demographics

In the 20-year period, 175 patients with esophageal adenocarcinoma underwent esophagectomy in our department. The median age was 62.6 years (range 28.9–79.9). There were 153 men (87.4%) and 22 women (12.6%). In the 10-year period between 9/1985 and 9/1995, 48 (27.4%) patients were resected for adenocarcinoma compared to 127 (72.6%) patients in the 10-year period between 10/1995 and 9/2005. At the time of the last follow-up in September 2006, 44/175 (25.1%) patients were alive. Median follow-up of the survivors was 37 months (range 6–170).

During the study period, 249 patients presented with squamous cell carcinoma; in 32 patients, an undifferentiated histologic type was found and 14 patients displayed other malignant tumors of the esophagus.

### Surgical therapy

123 (70.3%) patients underwent transhiatal esophagectomy and 52 (29.7%) had an abdominothoracic procedure with two-field (abdominal and mediastinal) lymphadenectomy.

Reconstruction was accomplished by pulled-up gastric tube in 168 (96.6%) patients, by colon interposition in 4 (2.3%), whereas in 2 (1.1%) patients no primary reconstruction was performed due to emergency resection (tumor haemorrhage). The anatomic prevertebral esophageal bed was used for the majority of these procedures (n = 165; 95.4%). Extra-anatomic reconstruction by the retrosternal route with cervical anastomosis after pull-up was carried out in 7 (4.0%) patients only. The 30-day mortality was 4.6% (8 patients).

### Long-term outcomes

Actuarial overall survival (R0-resections) was 33% at 3 years, 22.5% at 5 years, and 13.2% at 10 years, with a median survival of 21 months (range 0–170).

Multivariate analysis of prognostic factors for overall survival (R0-resections) proved pT-category (HR:1.574; 95%CI:1.220–2.031), nodal status (HR:1.790; 95%CI:1.136–2.820) and pM-category (HR:1.806; 95%CI:1.145–2.847) as independent predictors.

### Differences between time periods: 9/1985 to 9/1995 versus 10/1995 to 9/2005

Comparisons were made in demographics, tumor characteristics, and surgical treatment of the two time period groups in order to determine potential reasons for long-term outcome differences (Table 1). Significant differences were seen based on ASA-classification, tumor stage, and operative procedure (p < 0.05). There was no significant difference for age, gender, tumor site, residual tumor, and number of dissected or involved lymph nodes between time periods. 30-day mortality improved from 8.3% to 3.1% during the 20-year time-interval, thus without statistical significance.

**Table 1:** Clinicopathological features and operative course

	**Decade I (9/1985–9/1995) (n = 48)**	**Decade II (10/1995–9/2005) (n = 127)**	**p-value**
**age **(years)	62.1 (41–78.3)	62.6 (28.9–79.9)	n.s.

**gender **(males)	43 (89.6%)	110 (86.6%)	n.s.

**ASA-classification**			
-ASA I	0	0	
-ASA II	14 (30.4%)	58 (47.9%)	0.008*
-ASA III	27 (58.7%)	61 (50.4%)	
-ASA IV	5 (10.9%)	2 (1.7%)	

**tumor site**			
-middle third	8 (16.7%)	8 (6.3%)	n.s.
-lower third	40 (83.3%)	118 (93.7%)	

**UICC-classification**			
-I	6 (12.8%)	18 (14.3%)	
-IIA	9 (19.1%)	19 (15.1%)	0.017*
-IIB	0	24 (19%)	
-III	18 (38.3%)	30 (23.8%)	
-IV	14 (29.8%)	35 (27.8%)	

**R-classification**			
-R0	40 (83.3%)	115 (91.3%)	n.s.
-R1	6 (12.5%)	10 (7.9%)	
-R2	2 (4.2%)	1 (0.8%)	

**operative procedure**			
-transhiatal	16 (33.3%)	107 (84.3%)	<0.0001*
-transthoracic	32 (66.7%)	20 (15.7%)	

**number of lymph nodes**			
-dissected	19 (0–93)	23 (3–79)	n.s.
-involved	2 (0–76)	3 (0–33)	n.s.

**30-day mortality**	4 (8.3%)	4 (3.1%)	n.s.

*statistically significant

**Figure 1:**
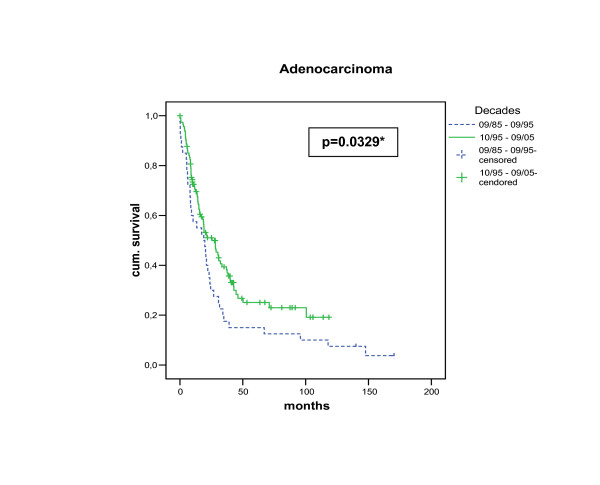
Comparison of two decades: Significantly better long-term survival for Decade II (10/1995 to 9/2005) as compared to Decade I (9/1985 to 9/1995) after curative (R0) resection for adenocarcinoma of the esophagus.

Differences in survival (R0-resections) between time periods are demonstrated in Figure 1. The overall survival was significantly more favourable in patients undergoing esophageal resection for adenocarcinoma in the recent time period compared to the early time period (log rank test: p = 0.0329). 3 (5)-year survival for this group improved from 17.5% (15%) to 40% (25%) between the two decades. Cox Proportional Hazard Model (R0-resections) for decade I revealed pN-category (HR:2.444; 95%CI:1.219–4.901) as the only independent prognostic factor of overall survival, whereas pT-category, distant metastases, grading, age, gender and ASA-classification were not significant (p > 0.05) (multivariate analysis). In contrast, decade II showed – in addition to pN-category (HR:1.866; 95%CI:1.005–3.462) – pT-(HR:1.777; 95%CI:1.249–2.529) and pM-category (HR:1.766; 95%CI:1.017–3.067) as independent predictors of favourable long-term survival with no significance for the other variables mentioned above (p > 0.05) (Table 2).

**Table 2:** Predictors of long-term survival: Cox Proportional Hazard Model(R0)

	**Decade I (9/1985–9/1995) (n = 40)**		**Decade II (10/1995–9/2005) (n = 115)**	
**multivariate analysis**	**Hazard Ratio (95% CI)**	**p-value**	**Hazard Ratio (95% CI)**	**p-value**

**pT-category**	1.267 (0.793–2.024)	n.s.	1.777 (1.249–2.529)	0.001*
**pN-category**	2.444 (1.219–4.901)	0.012*	1.866 (1.005–3.462)	0.048*
**pM-category**	1.544 (0.658–3.622)	n.s.	1.766 (1.017–3.067)	0.043*
**grading**	1.017 (0.593–1.746)	n.s.	1.143 (0.787–1.661)	n.s.
**age**	1.017 (0.970–1.067)	n.s.	1.002 (0.975–1.030)	n.s.
**ASA**	0.989 (0.460–2.125)	n.s.	0.993 (0.613–1.608)	n.s.
**gender**	1.882 (0.613–5.778)	n.s.	0.535 (0.235–1.217)	n.s.

*statistically significant

Subdividing all 175 patients into two groups dichotomized according to median overall survival (> 17.2 months: LTS = long-term survivors *versus *</= 17.2 months: STS = short-term survivors), significant differences in both decades were seen based on tumor stage only (p = 0.004 decade I and p > 0.0001 decade II) (Table 3).

**Table 3:** Long-term survival (LTS) *versus *Short-term survival (STS)

	**Decade I (9/1985–9/1995) (n = 48)**		**Decade II (10/1995–9/2005) (n = 127)**	
	**LTS/STS**	**p-value**	**LTS/STS**	**p-value**

**age (years)**	64.3/60.8	n.s.	61.9/63.3	n.s.

**ASA (%)**				
-ASA I	0		0	
-ASA II	28/33.3	n.s.	49.2/46.8	n.s.
-ASA III	60/57.1		49.2/51.6	
-ASA IV	12/9.5		1.7/1.6	

**operative approach (%)**				
-transhiatal	34.6/31.8	n.s.	88.7/80	n.s.
-transthoracic	65.4/68.2		11.3/20	

**UICC-classification (%)**				
-I	23.1/0		26.2/3.1	
-IIA	30.8/4.8		19.7/10.8	
-IIB	0	0.004*	23/15.4	<0.0001*
-III	23.1/57.1		16.4/30.8	
-IV	23.1/38.1		14.8/40	

**R-classification (%)**				
-R1	84.6/81.8	n.s.	93.5/89.1	n.s.
-R2	7.7/18.2		6.5/9.4	
-R3	7.7/0		0/1.6	

*statistically significant

## Discussion

This study, from a single tertiary-care referral center over a 20-year period, demonstrates significant developments in esophageal surgery for adenocarcinoma with regard to patient-related, tumor-specific and operative features and underlines our concept of surgical approach being offered to patients with this histologic entity.

Our data clearly show a favourable trend in improved long-term outcome over time for adenocarcinoma of the esophagus. Although significant advances in early detection, patient selection, operative technique and perioperative management have occurred in recent years, such a long-term trend in survival has not been well established in the literature. Several institutions have published their experience of improved outcome following resection of esophageal carcinoma including patients from different time periods [5-8]. Variability in inclusion criteria, neoadjuvant therapy, overlap of patient populations, and differences in analytic methods between reports limit the ability to draw conclusions regarding long-term outcome trends in survival from comparisons of these studies.

A variety of possible explanations for the observed trend in improved outcome for esophageal adenocarcinoma can be considered. Along with the changing epidemiology [9-12], surveillance programs for patients with Barrett's esophagus as well as better preoperative endoscopic and imaging studies play a critical role in improving patient selection [13-17]. Whereas early tumors were uncommon among the surgically treated patients with esophageal adenocarcinoma before 1990, early Barrett's carcinoma (pT1) now constitutes about 20% of all resected adenocarcinomas in our patients.

A recent report addressed an approximately 20% diagnostic gain with consecutive improved patient selection through better preoperative staging by positron emission tomography (PET) scan in combination with high resolution multislice CT scanning [18]. Due to the high sensitivity of CT findings with regard to metastatic sites in our patient population, preoperative PET was not characterized by greater accuracy of lesions previously detected by CT and consequently did not change the indication for esophagectomy [19]. Therefore, when determining the impact of newer staging techniques such as PET on long-term outcomes, carefully controlled trials should be considered.

The increasingly less aggressive surgical approach we have undertaken at our institution when confronted with adenocarcinoma of the esophagus – namely the transhiatal approach with a reduced perioperative morbidity and mortality as compared to transthoracic resection [20] – may also have contributed to the observed improvement in overall survival. Our concept of individualized surgical treatment according to the histologic type and extent of the disease is justified by findings derived from studies investigating the spread of lymph nodes in cases of adenocarcinoma, allowing one to conclude that the lymph node metastases associated with distal adenocarcinomas are initially seen to metastasize into the lymph nodes in the vicinity of the tumor and only later into the lymph nodes of the upper mediastinal region [21].

A recent analysis of own patients did not demonstrate a relevant difference in survival for patients with N0 and N1 stages undergoing transhiatal or transthoracic esophagectomy for adenocarcinoma [22]. It is questionable and has statistically not proved significant in a prospective randomized study by Hulscher et al. [23], if an extensive mediastinal lymph node dissection in addition to the clearance of abdominal lymph nodes offers any prognostic advantages in adenocarcinoma of the esophagus considering the increased morbidity associated with the transthoracic approach.

In the last decade, most experienced esophageal surgeons have implemented significant changes in operative technique for esophageal resection. Innovations in surgical technology, such as mechanical staplers and haemostatic devices have resulted in clear improvement in short-term outcomes. Several studies have reported trends in decreasing mortality and shorter hospital stay by improved perioperative management as peridural anesthesia, early extubation, and intensive physical therapy after esophageal resections. Whether advances in surgical technique and operative management contribute to improved long-term outcome is not clear.

Neoadjuvant chemoradiotherapy followed by surgery is being used with increasing frequency and continues to be actively studied in the surgical management of locally advanced esophageal cancer. Pathologic complete responses are seen in up to 30% of patients [24]. Thus, the beneficial effect of neoadjuvant therapy in esophageal adenocarcinoma remains doubtable. Our own long-term results are compared to randomized prospective trials including patients with adenocarcinoma treated either by chemotherapy plus surgery (CS) versus surgery alone (S) [25,26] or chemoradiotherapy plus surgery (CRT) versus surgery (S) [27-29] in Table 4. Only two of the listed trials could show a survival benefit of combined preoperative chemoradiotherapy [27] or preoperative chemotherapy [26]. However, survival analyses did not clearly differ between the two histologic tumor types and the single study consisting of patients with adenocarcinoma only [27], has largely been criticized for insufficient preoperative staging procedures and a very poor outcome of the surgery group with a 3-year-survival rate of 6%. A metaanalysis by Arnott et al. of all available trials concerning neoadjuvant radiotherapy – again without a clear differentiation between adenocarcinoma and squamous cell carcinoma – concluded that neoadjuvant radiotherapy did not improve survival and thus was not justified [30]. According to a critical appraisal recently published by SR DeMeester, a generic recommendation for neoadjuvant therapy in patients with esophageal adenocarcinoma is unwarranted, until complete pathologic response rates improve or until those patients most likely to achieve a complete response are accurately identified before initiation of therapy [31].

**Table 4:** Randomized prospective trials of neoadjuvant therapy plus surgery versus surgery alone for esophageal adenocarcinoma

**author/year**	**n**	**survival: C + S**	**survival: S**	**p-value**
**Kelsen/1998 (25)**	440 (54% ADC, 46% SCC)	14.9 mo 2 yr: 35%	16.1 mo 2 yr: 37%	n.s.
**MRC/2002 (26)**	802 (66% ADC, 31% SCC)	16.8 mo 2 yr: 43%	13.3 mo 2 yr: 34%	0.004*

		**survival: CR + S**	**survival: S**	

**Walsh/1996 (27)**	58 (100% ADC)	16 mo 3 yr: 32%	11 mo 3 yr: 6%	0.01*
**Urba/2001 (28)**	100 (75% ADC, 25% SCC)	16.9 mo 3 yr: 30%	17.9 mo 3 yr: 16%	n.s.
**Burmeister/2002 (29)**	256 (61.7% ADC, 37.1% SCC)	21.7 mo 3 yr: 38%	18.5 mo 3 yr: 31%	n.s.
**Junginger 2006**	175 ADC	17.2 mo 3 yr: 33%		

*statistically significant ADC: adenocarcinoma; SCC: squamous cell carcinoma

## Conclusion

In summary, our data provide significant developments in esophageal surgery for adenocarcinoma over a 20-year study period. Based on our experience, overall survival is improving over time. Factors that may play a role in this trend include early diagnosis and improved patient selection through better preoperative staging, improved surgical technique with a tailored approach carefully evaluated by physiologic patient status, comorbidity and tumor extent. Promising new horizons in the surgical treatment of esophageal adenocarcinoma are minimal-invasive and limited resections as well as molecularbiologic-based treatment modalities that will have to compete with the current achievements in the next decade.

## Competing interests

The author(s) declare that they have no competing interests.

## Authors' contributions

T.J., I.G. and F.S. initiated the present study, participated in its design and carried out the study. T.J. coordinated the study. I.G. and U.G. performed the collection of data and M.D. carried out the statistical analysis. I.G. drafted the first version of the manuscript and all authors read and approved the final manuscript.

## Pre-publication history

The pre-publication history for this paper can be accessed here:


